# Four-in-One: A Comprehensive Checklist for the Assessment of Pain, Undersedation, Iatrogenic Withdrawal and Delirium in the PICU: A Delphi Study

**DOI:** 10.3389/fped.2022.887689

**Published:** 2022-06-13

**Authors:** Monique van Dijk, Erwin Ista

**Affiliations:** ^1^Pediatric Intensive Care Unit, Department of Pediatric Surgery, Erasmus University Medical Center, Erasmus MC–Sophia Children's Hospital, Rotterdam, Netherlands; ^2^Department of Internal Medicine, Section Nursing Science, Erasmus University Medical Center, Rotterdam, Netherlands

**Keywords:** pain assessment, undersedation, distress, IWS, delirium, pediatric intensive care unit

## Abstract

**Objectives:**

Children's pain, undersedation, iatrogenic withdrawal syndrome and delirium often have overlapping symptoms, which makes it difficult to decide why a child in the PICU is not comfortable. Validated assessment tools for these conditions are available, but regular assessment with multiple instruments may be too time-consuming. Therefore, we aimed to develop a new holistic instrument–the mosaIC checklist–that incorporates the assessment of the four conditions.

**Materials and Methods:**

We conducted a two-rounds international Delphi study among experts working in PICUs worldwide to find cues that in combination or separately are relevant for the four conditions.

**Results:**

In the first Delphi round, 38 of the 48 enrolled participants (79%) completed a questionnaire; in the second round 32 of 48 (67%). Eventually, 46 cues in eight categories (e.g., facial, vocal/verbal, body movements, sleep /behavioral state, posture/muscle tone, agitation, physiological and contextual) were found relevant. Thirty-three (72%) were considered relevant for pain, 24 for undersedation (52%), 35 for iatrogenic withdrawal syndrome (76%) and 28 (61%) for pediatric delirium. Thirteen cues (28%) were considered relevant for all four conditions; 11's (24%) for only one condition.

**Conclusion:**

This Delphi study is the first step in developing a 4-in-1 comprehensive checklist to assess pain, undersedation, iatrogenic withdrawal syndrome and delirium in a holistic manner. Further validation is needed before the checklist can be applied in practice. Application of the mosaIC checklist could help determine what condition is most likely to cause a child's discomfort–and at the same time help reduce the PICU staff's registration burden.

## Introduction

Health care professionals became aware of the importance of pain assessment in hospitalized children in the 1990's, which resulted in the development of many different observational pain assessment tools (1), either for acute procedural pain, such as prick pain, or for use after major surgery and for prolonged pain.

Self-report tools are the gold standard for children aged 4 years and older who are able to communicate (2). The application of self-report tools is often not feasible in the pediatric intensive care unit (PICU), where typically two-thirds of the children are under the age of 4 years or being sedated. Thus, next to pain assessment, PICU staff often also need to assess the children's level of sedation. Because symptoms of pain and distress overlap, for instance body movements and hyper alertness, some tools have been validated for both conditions. Examples are the N-PASS (3, 4) and the COMFORT behavior scale (5, 6), both validated for different types of pain and level of (under)sedation. With regard to the prevalence of pain in children admitted to a PICU, prevalence data based on studies are not available to our knowledge. A systematic review from 2013 suggested that around 11% of PICU patients may suffer from undersedation (7).

Because children admitted to a PICU often receive benzodiazepines and/or opioids, they are at risk of iatrogenic withdrawal syndrome (IWS), especially after having consumed these drugs for more than 5 days. The most widely used tools to assess the risk of IWS are the Withdrawal Assessment-Tool-1 (8, 9) and the Sophia Observation Withdrawal Symptoms Scale (10, 11). The reported prevalence of IWS ranges widely from 5 to 87% (12), while a large prospective multi-center study in the US including > 1,000 patients found a prevalence of 47% (13).

Delirium in adult ICU patients has long been acknowledged, but not until in the early 2000's healthcare professionals acknowledged the existence of pediatric delirium as well (14–16), whereupon several assessments tools have been validated for infants and children (17–19). The estimated prevalence of pediatric delirium is 34% (range 17–66%, depending on the subgroup studied) (20). As for pain and undersedation, symptoms of IWS and pediatric delirium may considerably overlap (21, 22).

In clinical practice, application of four different instruments may be needed to find out why a child is uncomfortable, in order to be able to decide on the first line of treatment. Time-constrained staff may not be able to regularly apply four instruments may be challenging with. Even more so considering the shortage of ICU nurses in most countries in Europe including the yet unknown number of nurses leaving their profession due to the impact of the COVID-19 pandemic (23, 24). Still, a position paper from the European Society of Pediatric and Neonatal Intensive Care (ESPNIC) recommends that “validated assessment tools for pain, undersedation, withdrawal syndrome and delirium be integrated in pain treatment protocols” (25).

It has not gone unnoticed, however, that the behavioral cues associated with the different conditions overlap. A Venn diagram in the ESPNIC position statement showed which behavioral cues overlap between two to four of the conditions, and which cues are unique for a condition (25). The selected behavioral cues in this diagram were based on expert opinions, and had not yet been cross-validated with actual data. To fill this lacunawe performed a Delphi study among experts working in PICUs worldwide to determine which cues should be included in a new holistic instrument that is tentatively named the mosaIC checklist. The name refers to the phenomenon that a collection of different mosaic pieces, in this case the different adverse conditions, creates an overall picture that tells more than the individual elements. In this way, nurses could efficiently estimate which condition – pain, undersedation, IWS or delirium – is most likely present in an uncomfortable child and should be treated first. Another advantage would be avoiding the need of applying four assessment tools.

## Materials and Methods

This Delphi study was approved by the medical research ethics committee of the Erasmus University Medical Center, Rotterdam, the Netherlands (EMC 2021-0573). Participants completed an electronic consent form for every round in which they participated and were ensured of anonymity. This study was conducted according to the principles of the Declaration of Helsinki.

### Participants

We performed a modified two-round online Delphi study among international PICU experts from tertiary or quaternary PICUs between October 2021 and February 2022. The online Delphi format allowed us to reach a diverse group of international experts without compromising anonymity. Purposive sampling was based on predetermined criteria and the recruitment of experts from Asia, Australia, Europe, North America, and South America. Experts included PICU nurses, physicians, child psychiatrists or clinical researchers with a background in nursing or medicine. The predetermined inclusion criteria were both knowledge and practical experience with all four conditions. Applying a snowball sampling method, the members of our research group sent personal e-mails to colleagues, describing the aim of the Delphi study and expected time investment. In addition, they were asked to nominate other experts.

We aimed to include at least 30 participants, which sample size has been recommended to produce stable results and to enhance content validity (26).

### Procedure

We replaced the standard first round – usually consisting of initial open-ended questions and focus group discussions – with a literature search for validated assessment tools for pain, (under)sedation, IWS and delirium in PICU patients. Next, we extracted symptoms of pain, undersedation, IWS and delirium from these tools and categorized these as follows: Facial, Vocal/verbal, Body movements, Posture/muscle tone, Sleep and behavioral state, Agitation, Physiological items, and contextual factors (see **Table 2**).

Data were collected using the online survey tool LimeSurvey (27). In each of the two rounds, the potential participants were sent an information letter explaining the aim and content of that specific round, a consent form, the estimated time investment, and a deadline for completion. To optimize the response rate, we sent the potential participants a maximum of two reminders per round. Responses were anonymous both to the panel and research group.

In the first round, the participants were asked to rate the relevance of 39 symptoms for each of the four conditions: pain, (under)sedation, IWS, and delirium. Relevance was rated with a 9-point Likert scale from 1: absolutely irrelevant up to 9: absolutely relevant. Because ages of the PICU population range from 0 to 18 years, we also asked participants if they considered specific symptoms age-dependent. Further, they were invited to comment on included symptoms or to add other symptoms they considered relevant.

In the second round, participants were informed about the outcomes of the first round and invited to reconsider or confirm their opinion based on this information, and to rate newly added symptoms.

### Data Analysis

For each symptom, we calculated a median rating for relevance and a disagreement index (DI) for the four conditions separately (28). Median ratings between 7 and 9 were defined as relevant, 4 to 6 as somewhat relevant, and 1–3 as irrelevant. The DI was calculated by dividing the inter-percentile range (IPR) (IPR 0.3-0.7) by the IPR adjusted for symmetry (IPRAS). A DI below 1 was regarded as sufficient agreement (28). Finally, all symptoms for each condition were categorized by the combination of median score and the disagreement index. We distinguished three categories.

#### Category 1

The symptom is relevant (median of 7–9) and there is agreement within the panel (DI < 1). Items for which this held true in round 1 were not presented in the second round but directly included in the final checklist. This strategy served to reduce the burden for the participants.

#### Category 2

The symptom is relevant (median 7–9), however without agreement (DI ≥ 1), or the symptom is somewhat relevant (median 4–6) with agreement in the panel (DI < 1); if this in round 1 was the case for a particular symptom, the symptom was again presented in the second round.

#### Category 3

The symptom is somewhat relevant (median 4–6) without agreement (DI ≥ 1), or the symptom is irrelevant median 1–3) (with or without agreement in the panel). Symptoms ending in category 3 in both rounds will be discarded.

## Results

We enrolled 48 participants, predominately from Europe and South America (Table 1). In the first Delphi round, 38 of the 48 enrolled participants (79%) completed the questionnaire; in the second round 32 of 48 (67%). The majority of respondents in both rounds had more than 11 years of PICU experience, and all professions were represented.

**Table 1:** Demographic characteristics of the participants in the first and second Delphi rounds.

	**Round 1 (*N* = 38)**	**Round 2 (*N* = 32)**
Age, years^*^	45 (38.8–55)	45 (40.5–55)
Sex, female (*n*, %)	30 (79%)	24 (75%)
Profession		
• Nurse • Physician • Clinical researcher • Other	18 (47%) 12 (32%) 6 (16%) 2 (5%)	14 (44%) 10 (31%) 5 (16%) 3 (9%)
Years of work experience in PICU		
• 1–10 • 11–20 • > 20	14 (37%) 13 (34%) 11 (29%)	8 (25%) 13 (41%) 11 (34%)
Geographic area		
• Europe • Northern America • Southern America • Australia • Asia	24 (63%) 2 (5%) 9 (24%) 2 (5%) 1 (3%)	23 (72%) 0 6 (19%) 2 (6%) 1 (3%)

* *Median (IQR); IQR, interquartile range; PICU, pediatric intensive care unit*.

### Delphi Round 1

Table 2 presents the first-round median relevance scores per item for each condition, with between brackets the DI. Of the 46 items introduced by the researchers, only “skin color” was not selected in the final version. Participants suggested five extra items to include for the second round. That is, “wide open eyes,” “tongue out,” “staring look” in the category facial; “movements of arms and or legs” in the body movement category; and “flaccid/floppy” in de category posture/muscle tone.

**Table 2:** Panel median and disagreement index in rounds 1 and 2.

**Item**	**Round 1**				**Round 2**			
	**Pain**	**Sedation**	**IWS**	**Delirium**	**Pain**	**Sedation**	**IWS**	**Delirium**
**Facial**								
Grimacing	9 (0.1)	7 (0.5)	6 (0.6)	6 (0.9)			6 (0.5)	5.5 (1.0)
Frowning	8 (0.1)	6 (0.5)	6 (0.5)	5 (0.9)		6 (0.8)	6 (0.9)	4.5 (0.9)
Quivering chin	7 (0.2)	6 (0.9)	7 (0.2)	6 (0.9)		5 (1.0)		4.5 (1.0)
Deepened nasolabial fold	7 (0.4)	6 (0.3)	5 (0.9)	5 (0.9)		5 (0.8)	5 (0.9)	4.5 (0.9)
Wide open eyes^*^					7 (0.3)	7 (0.4)	6 (0.2)	7 (0.4)
Tongue out^*^					3 (0.4)	3.5 (0.6)	4 (0.9)	4 (1.0)
Staring look^*^					3.5 (0.4)	5 (0.9)	6.5 (0.5)	8 (0.1)
**Vocal/verbal**								
High pitched crying	8 (0.1)	6 (0.5)	8 (0.2)	6 (0.2)		6 (0.5)		6 (0.4)
Screaming	9 (0.1)	6.5 (0.6)	7 (0.4)	7 (0.2)		6 (1.7)		
Crying steadily	8 (0.3)	6 (0.5)	8 (0.4)	6.5 (0.4)		6 (0.5)		6 (0.2)
Sobbing or whining	8 (0.3)	7 (0.2)	7 (0.4)	6.5 (0.6)				6 (1.7)
Moaning	8 (0.1)	7 (0.4)	7 (0.4)	7 (0.4)				
Ouch	9 (0.1)	5 (0.9)	4.5 (0.9)	4 (0.9)		4 (1.0)	4 (1.0)	4 (1.0)
Incomprehensible speech	3.0 (0.4)	5 (0.9)	6.5 (0.7)	9 (0.1)		5 (1.7)	6 (0.4)	
**Body movements**								
Restlessness	7 (0.4)	8 (0.2)	8.5 (0.1)	8 (0.1)				
Kicking or legs drawn up	8 (0.3)	7 (0.4)	7 (0.4)	7 (0.4)				
Grasping specific area of the body	8.5 (0.3)	3 (1.0)	5.5 (1.7)	6 (1.6)			5 (1.4)	6 (0.5)
Tremors	6 (1.0)	5 (1.0)	9 (0.1)	8 (0.5)	6 (0.7)	5 (0.9)		
Response to stimuli	4.5 (0.9)	8 (0.5)	7 (0.4)	8 (0.3)	4 (0.8)			
Movements of arms and/or legs^*^					7 (0.2)	7 (0.0)	7 (0.0)	7 (0.2)
**Posture/muscle tone**								
Tense/rigid	8.5 (0.1)	6.5 (0.4)	7 (0.2)	7 (0.4)		7 (0.5)		
Clenched fists	8 (0.1)	6.5 (0.9)	7 (0.4)	6 (0.4)		8 (0.2)		6 (0.2)
Increased muscle tone	8 (0.3)	7 (1.0)	7 (0.2)	6 (0.9)		7 (0.2)		7 (0.4)
Flaccid/floppy^*^					2 (0.2)	7 (1.0)	3 (0.4)	6 (1.0)
**Sleep and behavioral state**								
Sleeplessness	7 (0.4)	7 (0.4)	8 (0.1)	8 (0.3)				
Hyperalert	6 (0.9)	6 (0.9)	8 (0.3)	8 (0.1)	7 (0.4)	7 (0.5)		
Awakens frequently	7 (0.4)	7 (0.4)	8 (0.2)	8 (0.3)				
Lack of attentiveness	5 (0.9)	5.5 (0.9)	7 (0.4)	9 (0.1)	5 (0.9)	5 (0.8)		
Lack of purposeful acting	4 (0.2)	4.5 (0.9)	7 (0.4)	9 (0.1)	4 (0.8)	5 (1.7)		
Lack of eye contact	4 (0.9)	5 (0.9)	7 (0.4)	9 (0.1)	4 (0.8)	6 (1.7)		
Hallucinations	2 (0.4)	3 (1.0)	8 (0.5)	9 (0)				
Disorientation	3 (0.4)	6 (0.9)	7.5 (0.4)	9 (0)		6 (1.0)		
Difficult to console	8 (0.3)	6 (0.9)	8 (0.3)	8 (0.2)		6 (0.9)		
**Agitation**								
Anxious	6.5 (0.2)	7 (0.4)	8 (0.3)	8 (0.3)	7 (0.4)			
Restlessness	8 (0.4)	7 (0.4)	8 (0.1)	8 (0.1)				
Irritability	7 (0.4)	7 (0.4)	8 (0.1)	8 (0.3)				
Fumbling	4 (0.5)	5.5 (0.9)	7 (0.4)	7 (0.4)	4 (1.4)	6 (0.4)		
**Physiological items**								
Increased heart rate	8 (0.1)	7 (0.2)	8 (0.2)	6 (0.5)				6 (0.5)
Tachypnea	8 (0.3)	6.5 (0.9)	7 (0.4)	6 (0.5)		6 (0.5)		6 (0.5)
Increased blood pressure	8 (0.1)	8 (0.4)	7 (0.2)	6 (0.5)				6 (1.0)
Low oxygen saturation	6 (0.5)	7 (0.7)	4 (0.9)	4 (0.9)	7 (0.6)		4 (1.6)	4 (0.5)
Skin color	6.5 (0.5)	6 (0.9)	6 (0.9)	4 (0.9)	6 (0.5)	4 (1.0)	6 (0.9)	4 (0.4)
Fever	4 (0.7)	4 (0.6)	8 (0.2)	5.5 (1.6)	4 (1.0)	3 (0.4)		4.5 (1.6)
Sweating	7 (0.4)	6 (1.7)	9 (0.1)	6 (0.6)		6.5 (1.0)		6 (0.2)
Vomiting	6 (0.9)	4 (0.9)	8 (0.1)	6 (0.9)	6 (0.5)	4 (1.0)		5.5 (1.0)
Diarrhea	3 (0.6)	3 (0.2)	8 (0.1)	5 (0.9)				4 (0.5)
**Other items/context**								
Major surgery	9 (0.1)	7 (0.6)	4 (0.9)	7 (0.4)			4 (0.5)	
Presence of Wounds	9 (0.1)	4 (0.9)	3 (0.2)	3.0 (0.6)		3.0 (0.2)		
Weaning opioids/benzodiazepines	7 (0.5)	7 (0.5)	9 (0.1)	8 (0.3)				
Weaning ventilation	4.5 (0.9)	7 (0.4)	6 (0.9)	6 (1.6)	3.5 (1.0)		6 (0.8)	
Acute onset / fluctuation of symptoms	6 (0.9)	5 (1.6)	6 (1.0)	8 (0.1)	6 (0.9)	4 (1.0)	6 (0.9)	

*Median (DI) – DI, disagreement index*;

* *new in round 2; IWS, iatrogenic withdrawal syndrome; green cells indicate relevant for that condition; orange cells indicate somewhat relevant; red cells indicate irrelevant*.

Thirty-nine per cent of the respondents considered the item “high-pitched” typically applicable to younger children. More than one-third of the respondents considered the items ouch, “incomprehensible speech,” “hallucinations,” and “disorientation” not applicable to young children.

### Delphi Round 2

Table 3 gives the end results of round 2, with 46 items distributed over the 8 categories of symptoms. The newly suggested items “tongue out” and “flaccid/floppy” posture in the first round were not considered sufficiently relevant in round 2. Thirty-three of the 46 items (72%) were considered relevant for pain, 24 for undersedation (52%), 35 for IWS (76%) and 28 (61%) for pediatric delirium. Thirteen items (28%) were considered relevant for all four conditions; three in the body movements category, two in the posture/muscle tone category, three in the sleep and behavioral state category, three in the agitation category, and one in the category others; i.e., weaning from opioids/benzodiazepines. Eleven items (24%) were considered relevant for only one condition; i.e., four for pain (frowning, deepened nasolabial furrow, saying ouch, grasping a specific area of the body); one for sedation (weaning of ventilation), three for IWS (fever, vomiting and diarrhea); and three for delirium (staring look, incomprehensible speech, and acute onset or fluctuation). Twenty-five of the 46 items (55%) were considered relevant for two or three conditions.

**Table 3:** Final MOSAIC checklist after round 2.

**Item**	**Pain**	**Under-sedation**	**IWS**	**Delirium**	**≠**
**Facial**					
Grimacing	✓	✓			2
Frowning	✓				1
Quivering chin	✓		✓		2
Deepened nasolabial fold	✓				1
Wide open eyes^*^	✓	✓		✓	3
Staring look^*^				✓	1
**Vocal/verbal**					
High pitched crying	✓		✓		2
Screaming	✓		✓	✓	3
Crying steadily	✓		✓		2
Sobbing or whining	✓	✓	✓		3
Moaning	✓	✓	✓	✓	4
Ouch	✓				1
Incomprehensible speech				✓	1
**Body movements**					
Restlessness	✓	✓	✓	✓	4
Kicking or legs drawn up	✓	✓	✓	✓	4
Grasping specific area of the body	✓				1
Tremors			✓	✓	2
Response to stimuli		✓	✓	✓	3
Movements of arms and/or legs^*^	✓	✓	✓	✓	4
**Posture/muscle tone**					
Tense/rigid	✓	✓	✓	✓	4
Clenched fists	✓	✓	✓		3
Increased muscle tone	✓	✓	✓	✓	4
**Sleep and behavioral state**					
Sleeplessness	✓	✓	✓	✓	4
Hyperalert	✓	✓	✓	✓	4
Awakens frequently	✓	✓	✓	✓	4
Lack of attentiveness			✓	✓	2
Lack of purposeful acting			✓	✓	2
Lack of eye contact			✓	✓	2
Hallucinations			✓	✓	2
Disorientation			✓	✓	2
Difficult to console	✓		✓	✓	3
**Agitation**					
Anxious	✓	✓	✓	✓	4
Restlessness	✓	✓	✓	✓	4
Irritability	✓	✓	✓	✓	4
Fumbling			✓	✓	2
**Physiological Items**					
Increased heart rate	✓	✓	✓		3
Tachypnea	✓		✓		2
Increased blood pressure	✓	✓	✓		3
Low oxygen saturation	✓	✓			2
Fever			✓		1
Sweating	✓		✓		2
Vomiting			✓		1
Diarrhea			✓		1
**Other items/context**					
Major surgery	✓	✓		✓	3
Presence of Wounds	✓	✓			2
Weaning ventilation		✓			1
Weaning opioids/benzodiazepines	✓	✓	✓	✓	4
Acute onset / fluctuation of symptoms				✓	1
Number of relevant items for each condition	33	24	35	28	

* *Newly included in Round 2*.

## Discussion

In this international Delphi study among PICU experts, we determined the content validity of a 46-item checklist–the mosaIC checklist–whose application is aimed at identifying the most likely condition (pain, undersedation, IWS or delirium) that causes a child's discomfort. Application of this 4-in-1 checklist could be especially useful in PICU patients who require prolonged mechanical ventilation or extra corporeal membrane oxygenation. or are admitted for a longer period for other reasons and receive benzodiazepines and opioids to endure the invasive treatments. Application of this checklist often may not be necessary for short-stay PICU patients.

With the exclusion of 11 items, 35 items showed overlap between two, three or all four conditions in question. The 13 items that were considered relevant for all four conditions have no real discriminatory value, but help to provide the bigger picture. The issue of overlapping symptoms and signs in the four conditions has been noted in earlier studies but primarily between pain and distress/agitation on the one hand and between IWS and PD on the other hand. We therefore validated the COMFORT-B scale for both pain and undersedation in the PICU population (5, 6). The combined use of the COMFORT-B scale for pain and sedation assessment was also included in the recent recommendations from the Society of Critical Care Medicine (SCCM) and the American Academy of Pediatrics (AAP) (29).

Madden et al. underlined the overlap in scoring systems for iatrogenic withdrawal syndrome and pediatric delirium (21). This apparent overlap was a reason for our team to develop and validate the SOS-PD (Sophia Observation withdrawal Symptoms-Pediatric Delirium scale) scale to assess both IWS and PD (17, 30).

We believe that application of the mosaIC checklist could help enhance clinical reasoning and avoid compartmental thinking (22). The four conditions in question are difficult to distinguish in children and application of the mosaIC may be conducive to open-mindedly and holistically assessment of children's discomfort.

There is a trend in the PICU community to introduce sedation protocols to avoid oversedation, IWS or pediatric delirium. A guideline published in 2022 by the Society of Critical Care Medicine addresses the need for the routine monitoring of pain, agitation, withdrawal, and pediatric delirium using validated tools (29). Because the application of four separate tools may be a burden for PICU staff, we propose that the mosaIC checklist might serve as an outcome measure in the future, notably because it allows obtaining a better picture of the uncomfortable child. Especially because the introduction of sedation protocols may not only impact level of sedation but also the risk of IWS and pediatric delirium (31, 32).

This is also the case with the introduction of the ABCDEF bundle [A: Assess, prevent, and manage pain; B: Both Spontaneous Awakening Trials (SATs) and Spontaneous Breathing Trials (SBTs); C: Choice of sedation; D: Delirium assessment, prevention, and management; E: Early mobility and exercise; and F: Family engagement and empowerment] in adult and pediatric ICU. This holistic approach of treatment seems in line with our suggested assessment method.

Still, how to apply the mosaIC in practice is yet to be determined. The relatively high number of items might be impractical in practice. A digital application including an algorithm seems the best approach to consider. Next, we need to study the measurement properties (e.g., interrater reliability, structural validity, and construct validity by hypothesis testing) according to the Consensus-based Standards for the selection of health Measurement Instruments (COSMIN) initiative (26). We also need to study if weighing of items is necessary to differentiate between less and more important items for each condition.

### Strengths and Limitations

A strength of our study is the fact that we received feedback from more than 30 experts across PICUs around the world in both the first and second round of our Delphi study.

Some limitations need to be addressed as well. For one thing, the mosaIC checklist needs to be further validated before it can be applied in practice. Furthermore, fewer experts from the US – two and none out of ten invited parties from the US - participated than we had hoped for, as they had been involved in many of the assessment instruments we analyzed for the first Delphi round. This is disappointing, too, because the limited input from the US might affect the willingness in future to use our instrument. Although we did not indicate in the Delphi study from which instrument a symptom was taken, some respondents may have been biased because they had experience with certain instruments. Another limitation was that we omitted to include the item “movement of arms and legs” in the list presented in the first round, as this item is part of a number of pain assessment tools. Fortunately, one of the experts added this item.

## Conclusion

One the challenges in PICUs worldwide is to understand what causes young patients' discomfort, especially when they are preverbal or not able to communicate due to, for instance, deep sedation s. After the introduction of pain and sedation assessment tools, we have seen the introduction of IWS assessment tools, while lately the management of pediatric delirium including its assessment has received much attention. We suggest that application of the mosaIC checklist could help PICU staff determine what condition is most likely to cause a child's discomfort which should be treated, accordingly and at the same time help reduce the PICU nurses' registration burden.

## Data Availability Statement

The raw data supporting the conclusions of this article will be made available by the authors, without undue reservation.

## Ethics Statement

The studies involving human participants were reviewed and approved by Medical Research Ethics Committee of the Erasmus University Medical Center, Rotterdam, Netherlands. The patients/participants provided their written informed consent to participate in this study.

## Author Contributions

Both authors listed have made a substantial, direct, and intellectual contribution to the work and approved it for publication.

## Conflict of Interest

The authors declare that the research was conducted in the absence of any commercial or financial relationships that could be construed as a potential conflict of interest.

## Publisher's Note

All claims expressed in this article are solely those of the authors and do not necessarily represent those of their affiliated organizations, or those of the publisher, the editors and the reviewers. Any product that may be evaluated in this article, or claim that may be made by its manufacturer, is not guaranteed or endorsed by the publisher.

## References

[1] Giordano V Edobor J Deindl P Wildner B Goeral K Steinbauer P. Pain and sedation scales for neonatal and pediatric patients in a preverbal stage of development: a systematic review. JAMA Pediatr. (2019) 173:1186–97. 10.1001/jamapediatrics.2019.3351 31609437

[2] Birnie KA Hundert AS Lalloo C Nguyen C Stinson JN. Recommendations for selection of self-report pain intensity measures in children and adolescents: a systematic review and quality assessment of measurement properties. Pain. (2019) 160:5–18. 10.1097/j.pain.0000000000001377 30180088

[3] Hummel PA Puchalski ML Creech SD Weiss MG. N-PASS: neonatal pain, agitation, and sedation scale - reliability and validity. In; *Pediatric Academic Societies' Annual Meeting*. Seattle (2003).

[4] Hummel P Puchalski M Creech SD Weiss MG. Clinical reliability and validity of the N-PASS: neonatal pain, agitation and sedation scale with prolonged pain. J Perinatol. (2008) 28:55–60. 10.1038/sj.jp.7211861 18165830

[5] van Dijk M de Boer JB Koot HM Tibboel D Passchier J Duivenvoorden HJ. The reliability and validity of the COMFORT scale as a postoperative pain instrument in 0 to 3-year-old infants. Pain. (2000) 84:367–77. 10.1016/S0304-3959(99)00239-0 10666543

[6] Ista E van Dijk M Tibboel D de Hoog M. Assessment of sedation levels in pediatric intensive care patients can be improved by using the COMFORT “behavior” scale. Pediatr Crit Care Med. (2005) 6:58–63. 10.1097/01.PCC.0000149318.40279.1A 15636661

[7] Vet NJ Ista E de Wildt SN van Dijk M Tibboel D de Hoog M. Optimal sedation in pediatric intensive care patients: a systematic review. Intensive Care Med. (2013) 39:1524–34. 10.1007/s00134-013-2971-3 23778830

[8] Franck LS Harris SK Soetenga DJ Amling JK Curley MA. The withdrawal assessment tool-1 (WAT-1): an assessment instrument for monitoring opioid and benzodiazepine withdrawal symptoms in pediatric patients. Pediatr Crit Care Med. (2008) 9:573–80. 10.1097/PCC.0b013e31818c8328 18838937 PMC2775493

[9] Franck LS Scoppettuolo LA Wypij D Curley MAQ. Validity and generalizability of the withdrawal assessment tool-1 (WAT-1) for monitoring iatrogenic withdrawal syndrome in pediatric patients. Pain. (2012) 153:142–8. 10.1016/j.pain.2011.10.003 22093817 PMC3254588

[10] Ista E de Hoog M Tibboel D Duivenvoorden HJ van Dijk M. Psychometric evaluation of the sophia observation withdrawal symptoms scale in critically ill children. Pediatr Crit Care Med. (2013) 14:761–9. 10.1097/PCC.0b013e31829f5be1 23962832

[11] Ista E van Dijk M de Hoog M Tibboel D Duivenvoorden HJ. Construction of the sophia observation withdrawal symptoms-scale (SOS) for critically ill children. Intensive Care Med. (2009) 35:1075–81. 10.1007/s00134-009-1487-3 19367394

[12] Best KM Boullata JI Curley MA. Risk factors associated with iatrogenic opioid and benzodiazepine withdrawal in critically ill pediatric patients: a systematic review and conceptual model. Pediatr Crit Care Med. (2015) 16:175–83. 10.1097/PCC.0000000000000306 25560429 PMC5304939

[13] Best KM Wypij D Asaro LA Curley MA. Randomized evaluation of sedation titration for respiratory failure study I. Patient, process, and system predictors of iatrogenic withdrawal syndrome in critically ill children. Crit Care Med. (2017) 45:e7–15. 10.1097/CCM.0000000000001953 27513532

[14] Turkel SB Braslow K Tavare CJ Trzepacz PT. The delirium rating scale in children and adolescents. Psychosomatics. (2003) 44:126–9. 10.1176/appi.psy.44.2.126 12618535

[15] Schieveld JN Leentjens AF. Delirium in severely ill young children in the pediatric intensive care unit (PICU). J Am Acad Child Adolesc Psychiatry. (2005) 44:392–4; discussion 5. 10.1097/01.chi.0000153231.64968.1a 15782087

[16] de Carvalho WB Fonseca MC. Pediatric delirium: a new diagnostic challenge of which to be aware. Crit Care Med. (2008) 36:1986–7. 10.1097/CCM.0b013e318176aeba 18520669

[17] Ista E van Beusekom B van Rosmalen J Kneyber MCJ Lemson J Brouwers A. Validation of the SOS-PD scale for assessment of pediatric delirium: a multicenter study. Crit Care. (2018) 22:309. 10.1186/s13054-018-2238-z 30458826 PMC6247513

[18] Traube C Silver G Kearney J Patel A Atkinson TM Yoon MJ. Cornell assessment of pediatric delirium: a valid, rapid, observational tool for screening delirium in the PICU. Crit Care Med. (2014) 42:656–63. 10.1097/CCM.0b013e3182a66b76 24145848 PMC5527829

[19] Smith HA Boyd J Fuchs DC Melvin K Berry P Shintani A. Diagnosing delirium in critically ill children: validity and reliability of the pediatric confusion assessment method for the intensive care unit. Crit Care Med. (2011) 39:150–7. 10.1097/CCM.0b013e3181feb489 20959783 PMC3776416

[20] Semple D Howlett MM Strawbridge JD Breatnach CV Hayden JC. A systematic review and pooled prevalence of delirium in critically ill children. Crit Care Med. (2022) 50:317–28. 10.1097/CCM.0000000000005260 34387241

[21] Madden K Burns MM Tasker RC. Differentiating delirium from sedative/hypnotic-related iatrogenic withdrawal syndrome lack of specificity in pediatric critical care assessment tools. Pediatr Crit Care Med. (2017) 18:580–8. 10.1097/PCC.0000000000001153 28430755

[22] Ista E van Dijk M. We can not compartmentalize our patients! overlapping symptoms of iatrogenic withdrawal syndrome, pediatric delirium, and anticholinergic toxidrome. Pediatr Crit Care Med. (2017) 18:603–4. 10.1097/PCC.0000000000001163 28574912

[23] Lopez V Anderson J West S Cleary M. Does the COVID-19 pandemic further impact nursing shortages? Issues Ment Health Nurs. (2022) 43:293–5. 10.1080/01612840.2021.1977875 34586955

[24] Cornish S Klim S Kelly AM. Is COVID-19 the straw that broke the back of the emergency nursing workforce? Emerg Med Australas. (2021) 33:1095–9. 10.1111/1742-6723.13843 34337873 PMC8420260

[25] Harris J Ramelet AS van Dijk M Pokorna P Wielenga J Tume L. Clinical recommendations for pain, sedation, withdrawal and delirium assessment in critically ill infants and children: an ESPNIC position statement for healthcare professionals. Intensive Care Med. (2016) 42:972–86. 10.1007/s00134-016-4344-1 27084344 PMC4846705

[26] Terwee CB Prinsen CAC Chiarotto A Westerman MJ Patrick DL Alonso J. COSMIN methodology for evaluating the content validity of patient-reported outcome measures: a Delphi study. Qual Life Res. (2018) 27:1159–70. 10.1007/s11136-018-1829-0 29550964 PMC5891557

[27] GmbH L,. LimeSurvey: An Open Source Survey Tool /LimeSurvey GmbH. Hamburg; LimeSurvey (2022). Available online at: http://www.limesurvey.org.

[28] Fitch K Bernstein SJ Aguilar MD Burnand B LaCalle JR Lazaro P. The RAND/UCLA Appropriateness Method User's Manual. Santa Monica: RAND (2001).

[29] Smith HAB Besunder JB Betters KA Johnson PN Srinivasan V Stormorken A. 2022 Society of critical care medicine clinical practice guidelines on prevention and management of pain, agitation, neuromuscular blockade, and delirium in critically ill pediatric patients with consideration of the ICU environment and early mobility. Pediatr Crit Care Med. (2022) 23:e74–110. 10.1097/PCC.0000000000002873 35119438

[30] Ista E Te Beest H van Rosmalen J de Hoog M Tibboel D van Beusekom B. Sophia observation withdrawal symptoms-paediatric delirium scale: a tool for early screening of delirium in the PICU. Aust Crit Care. (2018) 31:266–73. 10.1016/j.aucc.2017.07.006 28843537

[31] Shildt N Traube C Dealmeida M Dave I Gillespie S Moore W. “Difficult to sedate”: successful implementation of a benzodiazepine-sparing analgosedation-protocol in mechanically ventilated children. Children. (2021) 8:348. 10.3390/children8050348 33924822 PMC8146538

[32] Smith HAB Gangopadhyay M Goben CM Jacobowski NL Chestnut MH Thompson JL. Delirium and benzodiazepines associated with prolonged ICU stay in critically ill infants and young children. Crit Care Med. (2017) 45:1427–35. 10.1097/CCM.0000000000002515 28594681

